# Evaluation of community basic public health service effect in a city in Inner Mongolia Autonomous Region——based on entropy weight TOPSIS method and RSR fuzzy set

**DOI:** 10.1186/s13690-023-01151-x

**Published:** 2023-08-17

**Authors:** Xin Dai, Yiru Jiang, YingYing Li, XiaoXue Wang, Rongrong Wang, Yuanyuan Zhang

**Affiliations:** School of Public Health, Dalian Medical University, Dalian, Liaoning Province China

**Keywords:** Basic public health services, Technique for order preference by similarity to an ideal solution (TOPSIS), Rank-sum ratio (RSR), Fuzzy set

## Abstract

**Background:**

By analyzing 23 evaluation indicators included in 14 national basic public health service programs in a region of Hohhot City, Inner Mongolia Autonomous Region, China, the performance of basic public health services in the region in 2021 were analyzed to clarify the implementation and conduct of relevant programs. We also use this study as a basis to radiate the work of municipal basic public health services centered on the region and the outstanding problems reflected and to provide theoretical contents and suggestions that can be referred to for the same regions in central and western China as well as worldwide.

**Methods:**

Using the TOPSIS method as the basis for the data analysis method, the evaluation indexes are ranked in terms of their proximity to the idealized target, and combined with the entropy value method, Technique for order preference by similarity to an ideal solution (TOPSIS) and rank-sum ratio (RSR) were used to rank 14 basic health care providers by grade. A comprehensive evaluation of the performance of basic public health services in a region of Hohhot City, Inner Mongolia Autonomous Region in 2021 was conducted through a joint model of entropy -weighted TOPSIS and RSR, making full use of the characteristics and advantages of the fuzzy joint, and conducting a comprehensive analysis from the perspective of the ratio weight and the method of graded calculation, making the study more distinguishable and measureable.

**Results:**

In this study, for the regional basic public health services, a total of 23 evaluation indicators of basic public health service projects were included, among which the top three indicators with the weight of the entropy value method indicators were found to include the rate of Chinese medicine health management for the elderly, the rate of health management for the elderly, and the BCG vaccination rate after the analysis of the weight of the indicators; After the entropy-weighted TOPSIS evaluation showed that the Ci values of the regions were found to be between 0.378 and 0.715 through the calculation of the positive and negative ideal values of each indicator; RSR staging method evaluation showed that three community health centers (X2, X10, X12) had excellent evaluations of basic public health services; The number of evaluations as poor and moderate are 2 (X3, X9) and 9 (X1, X4, X5, X6, X7, X8, X11, X13, X14), respectively; Finally, the results of the entropy-weighted TOPSIS method and the fuzzy joint model of RSR staging method are basically consistent with the overall trend of the above two methods, and the reliability and credibility of the research results are high.

**Conclusion:**

The entropy-weighted TOPSIS and RSR joint model can evaluate the effectiveness of basic public health services in a more comprehensive and holistic way. The results of the RSR staging results and the related weight ratio analysis show that the basic public health service programs in Hohhot, Inner Mongolia Autonomous Region are relatively balanced, but there are some differences; The same genus of elderly Chinese medicine health management rate, health management rate of the elderly, BCG vaccination rate several indicators accounted for a higher weight, its correlation with the key population-related items is high, suggesting that the future key population health service items should be focused on, and future research should be suggested from two key research.

**Table:** 

**Text box 1. Contributions to the literature**	
• The fuzzy joint model of entropy right TOPSIS and RSR evaluates the effect of basic public health service from the perspective of ratio weight and classification calculation, the method reliability and credibility are relatively high; • The results show that the degree of policy tilt and economic development is directly proportional to the quality of basic public health services, and the importance of decision-making at the strategic level should be concerned; • From the demographic characteristics, economy, space and other factors, focus on improving the awareness rate and utilization of health service projects of special groups and their family members.	

## Introduction

The "National Basic Public Health Service Project" is an important part of China's public health system construction. The project provides basic medical and health services as public goods to the whole people, and institutionalizes them to improve and extend them. In 2021, China raised the national per-capital funding standard for basic public health services to 70 yuan. Basic public health service items include 56 items in 13 categories, including the establishment of resident health records, maternal health management, and vaccination for urban and rural residents free of charge [1].

At present, there are many evaluation methods for the effectiveness of basic public health services at home and abroad. The World Health Organization uses generalized cost–benefit analysis (GCEA) to evaluate the average cost–benefit ratio (ACER) [2] to measure the efficiency of the health system; low- and middle-income countries have also widely introduced social responsibility programs and improved public health services in health institutions [3], Fox [4], Ogbuabor & Onwujekwe [5], Joshi [6] believe that social responsibility plans can be practiced through scorecards, community scorecards, and formal public disclosure of data by community health facility committees. It is rare to use the combined model of TOPSIS and RSR based on entropy weight to evaluate the effectiveness of community health services. The TOPSIS method is a technique for order preference based on similarity to the ideal solution. The method is based on normalizing the raw data matrix to form a space for the positive and negative ideal solutions of the preferred solution [7]. This method essentially judges the advantages and disadvantages of each scheme in the data based on the existing data when there are multiple indicators and multiple schemes [8]. The TOPSIS method has a wide range of applications and is generally used in corporate management, marketing, investment decision-making, and other fields. Luo Huahua and Liu Jianhua used TOPSIS to explore the factors that affect Malaysia's adoption of medical tourism [9], and determined the importance of the influencing factors. The RSR method is a comprehensive evaluation and analysis method proposed by Chinese statistician Professor Tian Fengtiao in 1988 [10]. The RSR method is more used in the field of evaluating the quality of hospital medical work [11]. Shen Minxue, Zeng Na, and others used the RSR method to evaluate the rationality and effectiveness of drug supervision and supply network construction in rural areas of Hunan Province [12].

In order to carry out basic public health services in a solid and orderly manner, Hohhot City, Inner Mongolia Autonomous Region, has set up special public health departments or public health service project management departments with the banners, counties, districts, and grass-roots medical and health institutions as units. These management departments decompose the project tasks step by step, and incorporate the implementation results into the local government's performance assessment management and assessment plan to actually implement the basic public health service project work [13]. The appraisal was conducted according to “the Performance Evaluation Program of the Basic Public Health Services Program for 2021”.This study takes a deeper and more refined perspective on the development of basic public health service programs in provincial and municipal districts. This study uses the entropy-weighted TOPSIS method in conjunction with the RSR staging model to conduct a comprehensive evaluation of the performance of primary healthcare services in each district, to provide a scientific basis for promoting the construction of basic public health services in provincial and municipal areas in China. The study focused on the municipal regional level, targeting all citizens, to implement health interventions for basic public health problems that have a general and serious impact problems that have a general and serious impact on the health of the population, and strive to apply limited resources to the problems most closely related to the health of the population, so that the work of basic public health programs can achieve the best results [14], to provide an effective reference for the implementation of basic public health service programs in the same type of urban areas in China and internationally.

## Data sources

### Source

This study was derived from the 2021 annual report data of public health service project performance assessment in Hohhot City, Inner Mongolia Autonomous Region, provided by the Health and Health Commission of Hohhot City, Inner Mongolia Autonomous Region. Based on the economic development level and population size of each banner and county district, 14 primary health care service centers in a district were selected by the method of intentional sampling through field visits, inspections, and the 2021 annual report data of the performance assessment of basic public health services 14 community health service centers., were selected for the study, including the community health service centers and township health centers, denoted by X1-X14.^1^

### Indicator determination

According to the content of "2018 National Basic Public Health Service Project" [15]. issued by the Chinese Health Commission, 23 indicators were selected as evaluation indicators, including: TCM health management rate of the elderly, TCM health management service rate for children aged 0–36 months, Early pregnancy registration rate, Postpartum visits, Hepatitis B vaccination rate, BCG vaccination rate, Polio vaccination rate, DTP vaccination rate, Vaccination rates containing measles components, Meningococcal vaccination rate, JE vaccination rate, Hepatitis A vaccination rate, Standardized management rate of patients with severe mental disorder, Elderly health management rate, Standardized management rate of patients with type 2 diabetes, Manage population glycemic control rate, Standardized management rate of hypertensive patients, Manage population blood pressure control rate, Tuberculosis patient management rate, The regular medication rate of pulmonary tuberculosis patients, Electronic health record filing rate, Neonatal visits, and Child health management rate.

## Method

In the evaluation of the quality of basic public health services, in order to ensure the balance of data To avoid the influence of the data itself on the evaluation results, the TOPSIS method is used in combination with the RSR method. Combining the TOPSIS method with the RSR method can not only avoid the disadvantage of the TOPSIS method being susceptible to discrete values, but also better represent the characteristics of the data. This also emphasizes the importance of different evaluation indicators. Compared with using the TOPSIS method or RSR alone The fuzzy combination of the two methods is used for comprehensive evaluation, which can make full use of the characteristics and advantages of fuzzy combination, and conduct comprehensive analysis from the perspective of ratio weight and classification calculation method, making the research more distinguishable and measureable. The statistical analysis of relevant data was completed using Excel 2016 and SPSS 22.0 software.

### Entropy value method

The smaller the entropy value, the more information the indicator provides and the greater the weight; conversely, the smaller the weight. Compared with subjective methods such as Delphi method and hierarchical analysis, the entropy method depends entirely on the data itself, which avoids the influence of subjective factors and is more objective.

1. Standardization of data. Since all the indicators involved in this study are positive indicators, i.e., highly superior indicators. Therefore, the formula of positive indicators is used to standardize the data, which is

1 $${r}_{ij}={x}_{ij}-{\left({x}_{ij}\right)}_{\mathrm{min}}/{\left({x}_{ij}\right)}_{\mathrm{max}}-{\left({x}_{ij}\right)}_{\mathrm{min}}$$

(2) Determine the weights of the indicators. The weight of the jth indicator is

2 $${w}_{j}=\left(1-{E}_{j}\right)/\left(n-\sum_{i=1}^{n}\left({P}_{ij}\text I\mathrm{n}{P}_{ij}\right.\right)$$

Among

3 $${E}_{\mathrm{j}}=\left(-1\right./\left.\text I\mathrm{n}m\right)\sum_{i=1}^{m}\left({P}_{ij}\text I\mathrm{n}{P}_{ij}\right)$$

is called the information entropy of the jth indicator,

4 $${P}_{\mathrm{ij}}={r}_{ij }/\sum_{i=ij}^{m}{r}_{ij}$$

is the characteristic weight of the jth indicator.

### TOPSIS

The conceptual framework of the TOPSIS method was developed by Hwang and Yoon [16]. As a decision-making method with strong applicability, the entropy weight TOPSIS method is suitable for multi-objective decision-making processing, and its principle and application are simpler in practical application than other evaluation methods [7]. The entropy weight TOPSIS method makes full use of the original data for evaluation and research, which is convenient to operate clearly and distinctly shows the gap between the various objectives, and the obtained results have the advantage of comparability. The basic steps are as follows [17].

1. Indicator consistency: Convert low excellent indicators into high excellent indicators. Since the data indicators selected in this study are all high excellent indicators, the indicator conversion steps are omitted;
2. The original data which

5 $$\mathrm{X}=\left[\mathrm{Xij}\right]=\begin{array}{c}A1\\ A2\\ \vdots \\ An\end{array}\left[\begin{array}{cccc}x11& x12& \cdots & x1m\\ x21& x22& \cdots & x2m\\ \cdots & \cdots & \cdots & \cdots \\ xn1& xn2& \cdots & xnm\end{array}\right]$$

is normalized by

6 $${a}_{ij}={X}_{ij}/\sqrt{{\sum }_{i=1}^{n}{X}_{ij}^{2}}$$

(3) Determine the best plan and the worst plan, where the best plan (extreme performance for each metric):

7 $${a}^{+}=\left({a}_{i1}^{+},{a}_{i2}^{+},...,{a}_{im}^{+}\right)$$

and the worst plan (reverse extreme performance for each metric):

8 $${a}^{-}=\left({a}_{i1}^{-},{a}_{i2}^{-},...,{a}_{im}^{-}\right)$$

(4) Calculate the best plan and the worst plan of the $i$ evaluation object, and the expressions are as follows, *i*

9 $${D}_{i}^{+}= \sqrt{\sum\nolimits_{j=1}^{m}{\left({a}_{{ij}^{-}}^{+}-{a}_{ij}\right)}^{2}}$$

10 $${D}_{i}^{-}= \sqrt{\sum\nolimits_{j=1}^{m}{\left({a}_{{ij}^{-}}^{-}-{a}_{ij}\right)}^{2}}$$

(5) Calculate the final evaluation index of the concrete performance of each evaluation object, that is, the approach degree *C*_*i*_ between the *i* evaluation object and the optimal plan, and its mathematical expression is *iC*_*i*_

11 $${C}_{i}={D}_{i}^{-}/{\left({D}_{i}^{+}+ {D}_{i}^{-}\right)}_{\left(i=1,2,...,n\right)}$$

*C*_*i*_ is bounded between 0 and 1, The smaller $D_{i}^{ + }$ is, the higher the weight of an indicator and, therefore, the larger it is. On the contrary, a small ${D}_{i}^{-}$ indicates that an indicator has a lower weight and a lower weight ratio ranking [18].

### RSR

The core concept of the RSR method is to combine multiple indicators of the evaluation object into a statistic between 0 and 1 with the characteristics of linked variables [19]. The dimensionless statistic RSR is obtained by rank transformation, and on this basis, the distribution characteristics of RSR are studied, and the statistically significant RSR regression equation can be used to describe the degree of superiority or inferiority of the evaluation object [20]. The basic steps of the RSR method are as follows:

1. Rank the evaluation objects. Organize the evaluation objects into an original data matrix with n rows and m columns; Compile the order (rank) of each type of evaluation index in different evaluation objects, and sort the high-quality indicators in the order from small to large, and vice versa;
2. Calculate: The calculation equation is.

12 $${RSR}_{i}={\sum }_{j=1}^{m}\frac{{R}_{ij}}{mn}$$

By calculating and comparing the value of *RSR*, the evaluation objects are preliminary sorted comprehensively; When there are too many evaluation objects, the distribution of *RSR* to be found out is sorted by grades;

(3) Calculate the probability unit (Probit) value. The sorted RSR values are sorted from small to large (those with the same value are a group), Compile the RSR frequency distribution table, calculate the frequency (f) and the cumulative frequency of each group, and convert the cumulative frequency into the probability unit value with reference to the "Percentage and Probability Unit Comparison Table" [21].
(4) Taking the probability unit value (Probit) as the independent variable and RSR as the dependent variable, calculate the linear regression equation.
(5) The evaluation objects are sorted according to the RSR value, and the sorting is generally divided into 3–5 grades.

### Fuzzy set of TOPSIS and RSR

Using FUZZY SET theory [22], set the weight ratio of *C* value to *RSR* value *W*_*1*_: *W*_*2*_, that is, find.

13 $${W}_{1}C+{W}_{2}\times RSR$$

According to the values of *W*_*1*_ and *W*_*2*_, it can be divided into several grades, for example, *C*: *RSR* is 1: 0, 0.1: 0.9, 0.5: 0.5, 0.9: 0.1 and 0: 1 respectively. According to the "most principle", there are $n$ models

14 $$\underset\sim{A_{1}}, \underset\sim{A_{2}},\dots \underset\sim{A_{n}}\in \oint \left(U\right)$$

and each

15 $$\underset\sim{A_{n}}\left(n=1,2,...,n\right)$$

has a feature set expressing a certain attribute. If $\underset\sim{A_{1}}$ with the same feature set is divided into one group, which can be divided into $m$ group, 1 ≤ *m* ≤ *n,* then the feature set containing the most $\underset\sim{A_{i}}$ in each group is selected, and the feature set is the ranking set of various comprehensive evaluations. Obviously, *m* = 1 is a special case. In, *m* = *n* it shows that the feature set is too scattered, so we have to expand $\underset\sim{A_{i}}$ and then group it again until the feature set is concentrated.

## Results

### Entropy weight TOPSIS index weight

The 23 indexes selected in this study are all high and excellent indexes (positive indexes), so the index conversion step is omitted. By standardizing the positive indexes by,

16 $${b}_{ij}= \frac{{a}_{ij }- {a}_{j}^{\mathrm{min}}}{{a}_{j}^{\mathrm{max}}-{a}_{j}^{\mathrm{min}}}$$

the weights of basic public health service evaluation indexes under entropy weight method are calculated [23]. The top 5 weight ratios are the health management rate of traditional Chinese medicine for the elderly, the health management rate of the elderly, the BCG vaccination rate, the blood sugar control rate of the management population, and the meningococcal vaccination rate (Table 1).

**Table 1:** Entropy power TOPSIS index weight of 23 public health service indicators in a city in Inner Mongolia Autonomous Region in 2021

Indicators	Entropy value	Weights	Rank
TCM health management rate of the elderly(%)	0.8301	9.54%	1
TCM health management service rate for children aged 0–36 months (%)	0.8893	6.22%	6
Early pregnancy registration rate (%)	0.9707	1.64%	22
Postpartum visits (%)	0.9712	1.62%	23
Hepatitis B vaccination rate (%)	0.9452	3.08%	14
BCG vaccination rate (%)	0.8708	7.26%	3
Polio vaccination rate (%)	0.9262	4.15%	11
DTP vaccination rate (%)	0.9151	4.77%	10
Vaccination rates containing measles components (%)	0.9325	3.79%	12
Meningococcal vaccination rate (%)	0.8863	6.39%	5
JE vaccination rate (%)	0.9147	4.79%	9
Hepatitis A vaccination rate (%)	0.9506	2.78%	16
Standardized management rate of patients with severe mental disorder (%)	0.9482	2.91%	15
Elderly health management rate (%)	0.8675	7.44%	2
Standardized management rate of patients with type 2 diabetes (%)	0.9508	2.76%	17
Manage population glycemic control rate (%)	0.8742	7.07%	4
Standardized management rate of hypertensive patients (%)	0.9512	2.74%	18
Manage population blood pressure control rate (%)	0.9375	3.51%	13
Tuberculosis patient management rate (%)	0.9676	1.82%	21
The regular medication rate of pulmonary tuberculosis patients (%)	0.9087	5.13%	8
Electronic health record filing rate(%)	0.9529	2.64%	19
Neonatal visits(%)	0.8923	6.05%	7
Child health management rate(%)	0.9659	1.92%	20

### Entropy weight TOPSIS comprehensive evaluation results

Firstly, the weight value of each evaluation index is calculated by the entropy weight method, the indexes in the standardized matrix are weighted, and the weight is multiplied by the normalized index value to form a weighted matrix, and the positive and negative ideal solutions of each evaluation index are obtained [24]. Then calculate the relative progress and priority of each object to be evaluated (Table 2). The results show that the top three basic medical and health service institutions include X10, X13 and X2, and the bottom three basic medical and health service institutions include X5, X6 and X9, which is closely related to the results of the entropy value method after calculating the weights of each indicator. That is to say, among the 14 basic medical and health service institutions, the institutions with a large proportion of the weight of 23 service indicators and the development of basic public health service projects are relatively high.

**Table 2:** Calculated results of TOPSIS evaluation of the effectiveness of basic public health implementation in 14 districts in a city of Inner Mongolia Autonomous Region in 2021

Rank	District	$\boldsymbol D_{\mathbf i}^{\boldsymbol{+}}$	$\boldsymbol D_{\mathbf i}^{\boldsymbol{-}}$	*Ci*
1	X10	0.077	0.193	0.715
2	X13	0.112	0.159	0.586
3	X2	0.119	0.158	0.572
4	X1	0.121	0.157	0.566
5	X12	0.139	0.153	0.524
6	X7	0.138	0.152	0.523
7	X11	0.154	0.139	0.474
8	X8	0.145	0.117	0.447
9	X14	0.154	0.12	0.438
10	X4	0.16	0.108	0.403
11	X3	0.179	0.113	0.386
12	X5	0.171	0.105	0.382
13	X6	0.171	0.101	0.372
14	X9	0.183	0.081	0.308

### RSR sorting and grading results

The index values in Table 1 are ranked, and the high-quality indexes are ranked from small to large; Calculate the value of $RSR$ by integer method, and get the frequency *f* and average rank *R* of each distribution value; According to the average rank value,

17 $$R/n\times 100\%,$$

the probability distribution Probit value [25]. is calculated in Table 3. Taking the value of $RSR$ in the table as independent variables and the Probit value as dependent variables, the regression equation is calculated as: distribution value of *RSR* = -0.132 + 0.130 $\times$ Probit (F = 368.9643, *p* < 0.01), which shows that the difference between the equations is statistically significant and the fitting degree is high..

**Table 3:** Frequency distribution of RSR values and probit in 14 districtsin

Districts	*RSR*	*f*	*R*	R/n*100%	*Probit*
X1	0.5824	1	8	57.143	5.18
X2	0.6468	1	12	85.714	6.068
X3	0.352	1	1	7.143	3.535
X4	0.4574	1	6	42.857	4.82
X5	0.4017	1	3	21.429	4.208
X6	0.4232	1	4	28.571	4.434
X7	0.6082	1	10	71.429	5.566
X8	0.5259	1	7	50.000	5
X9	0.3565	1	2	14.286	3.932
X10	0.7811	1	14	100.000	7.1
X11	0.5963	1	9	64.286	5.366
X12	0.6274	1	11	78.571	5.792
X13	0.6854	1	13	92.857	6.465
X14	0.4557	1	5	35.714a	4.634

A corrected by (1–1/4*n)*100%

The performance evaluation of basic public health services in 14 districts is divided into 3 grades, of which X2, X10, and X13 are good grades, X3 and X9 are poor grades, and the remaining 9 are medium grades (Table 4). The results of this analysis also reaffirm the results of the entropy TOPSIS method above, and the results of both studies are highly consistent.

**Table 4:** Basic public health service performance evaluation staging results for 14 districts ofa city in Inner Mongolia Autonomous Region in 2021

Level	Percentile critical value	Probit critical value	RSR critical value	Grading Results
poor	< 15.866	< 4	< 0.387	X3, X9
Medium	15.866 ~ < 84.134	4 ~ < 6	0.387 ~ < 0.646	X1, X4, X5, X6, X7, X8, X11, X12, X14
Outstanding	≥ 84.134	≥ 6	≥ 0.646	X2, X10, X13

Using the FUZZY SET theory, the entropy weight TOPSIS and the RSR fuzzy set have adopted three different distribution ratios: (1) *C*_*i*_: *RSR* = 0.1: 0.9;(2) *C*_*i*_: *RSR* = 0.5: 0.5;(3) *C*_*i*_: *RSR* = 0.9: 0.1 (Table 5). After fuzzy union of the entropy-weighted TOPSIS method and the RSR method, the results of Union 1 show that the top three institutions are X10, X13, and X2; the results of Union 2 show that the top three institutions are X10, X13, and X2; the results of Union 3 show that the top three institutions are X10, X13, and X2.

**Table 5:** The fuzzy sets of entropy TOPSIS and RSR

Districts	entropy TOPSIS		RSR		The fuzzy sets of entropy TOPSIS and RSR					
	Ci	rank	RSR	rank	0.1C + 0.9RSR	rank	0.5C + 0.5R	rank	0.9C + 0.1RSR	rank
X1	0.5656	4	0.5824	7	0.58072	7	0.574	5	0.56728	4
X2	0.5719	3	0.6468	3	0.63931	3	0.60935	3	0.57939	3
X3	0.3864	11	0.352	14	0.35544	13	0.3692	13	0.38296	12
X4	0.4026	10	0.4574	9	0.45192	10	0.43	10	0.40808	10
X5	0.3817	12	0.4017	12	0.3997	12	0.3917	12	0.3837	11
X6	0.3717	13	0.4232	11	0.41805	11	0.39745	11	0.37685	13
X7	0.5234	6	0.6082	5	0.59972	5	0.5658	6	0.53188	6
X8	0.4466	8	0.5259	8	0.51797	8	0.48625	8	0.45453	8
X9	0.3079	14	0.3565	13	0.35164	14	0.3322	14	0.31276	14
X10	0.7154	1	0.7811	1	0.77453	1	0.74825	1	0.72197	1
X11	0.4742	7	0.5963	6	0.58409	6	0.53525	7	0.48641	7
X12	0.5244	5	0.6274	4	0.6171	4	0.5759	4	0.5347	5
X13	0.5855	2	0.6854	2	0.67541	2	0.63545	2	0.59549	2
X14	0.4383	9	0.4557	10	0.45396	9	0.447	9	0.44004	9

## Discussion

### Analysis of influencing factors of TOPSIS results

From the entropy weight TOPSIS method to analyze the index weight, the overall distribution is relatively balanced and stable. However, the health management rate of Chinese medicine (9.54%), the health management rate of the elderly (7.44%), the BCG vaccination rate (7.26%), the blood sugar control rate of the management population (7.07%), the epidemic cerebrospinal meningitis vaccination rate (6.39%) and the newborn visit rate (6.05%) accounted for a higher proportion. There are some differences, which shows that there are some difficulties in the health management of the elderly, the management effect of diabetic patients, and the systematic healthmanagement and vaccination of children in this area [26].

Through the analysis results of TOPSIS method, it is concluded that most of the primary medical and health institutions in Hohhot, Inner Mongolia Autonomous Region, can carry out main basic public health services, but there is a certain gap in service quality. From the TOPSIS evaluation results, it can be seen that there are differences in the quality of basic public health services in 2021 among the 14 community health service centers in the district. The average ${\mathrm{C}}_{\mathrm{i}}$ value of the 14 primary medical institutions is 0.478, and the ${\mathrm{C}}_{\mathrm{i}}$ value of X10, which ranks first, is 0.715. It is quite different from the ${\mathrm{C}}_{\mathrm{i}}$ value of X9 in the last digit (0.308). The ${\mathrm{C}}_{\mathrm{i}}$ value of X10 ranked first is 0.715, and the ${\mathrm{C}}_{\mathrm{i}}$ value of X9 ranked last is only 0.308, which is about 2 times apart. It can still be shown that there are regional differences in basic public health services between regions, and the equalization of basic public health services in regions needs to be improved. Combining the entropy weight method to calculate the weights of 15 types of basic public health service project indicators, the results show that because some public health services were promoted and standardized management after the introduction of the "Basic Public Health Service Projects" in 2009, while some public health services were both popularized and standardized. The results of calculating the weights of 23 basic public health service indicators with the entropy weighting method show that the degree of variation between the indicators of different basic public health service items is greater due to the time of development and the degree of standardization of some services that are relatively high.

### Discussion on sorting results of RSR method

The basic principle of RSR comprehensive evaluation method is to obtain dimensionless statistical value through rank transformation. The larger the RSR, the better the work in this area of Hohhot, Inner Mongolia Autonomous Region [27]. The results showed that the highest value of *RSR* of X10 was 0.7811, and the lowest value of *RSR* of X3 was 0.352 in 14 community health service centers, suggesting that the basic public health work of X10 was better, while the basic public health service capacity of X3 needed to be strengthened and improved. According to each index and the actual situation, it can be divided into three grades: poor score (< 0.387), medium score (0.387–0.646) and excellent score (≥ 0.646), and the feasibility of comprehensive evaluation is high. X2, X10 and X13 belong to the better gear, while X3 and X9 belong to the worse gear. X2 and X10 belong to areas with relatively mature economic development and more than 90% awareness rate of public health services.

After classifying the evaluation results with the RSR method, it was found that the development of basic public health services was affected due to objective reasons such as historical development or different economic and policy inclinations. Compared with other regions, X10 has obvious advantages in government support and more policy inclinations, which is conducive to promoting the improvement of the quality of basic public health service data and can better reflect the work effect. The geographical scale and population of X3 are relatively small, and its status is not prominent in the previous research on health resource allocation, and the management of grass-roots health is relatively difficult. X9 is the lack of capacity building of the grass-roots medical and health system. Therefore, relevant departments should not only implement the investment of funds, but also give full play to the effectiveness of funds to improve residents' sense of access to health services. It is necessary to fully implement the personal responsibility for the health of residents in the region, and advocate for them to develop a healthy way that fits the characteristics of their personal lives. While ensuring that basic public health services are provided to them, individuals can also use the basic public health services provided to ensure a balance between supply and demand of services.

### Fuzzy combined comprehensive evaluation of TOPSIS and RSR

According to the entropy weight TOPSIS comprehensive analysis results, the *C*_*i*_ average value of 14 community health service centers in this area of Hohhot City is 0.478, 6 township hospitals are higher than the average value, and 8 township hospitals are lower than the average value. Among them, the highest value *C*_*i*_ of X10 is 0.715, and the lowest value *C*_*i*_ of X14 is 0.308. The *C*_*i*_ of difference between X10 and X14 is large, which shows that there is a big gap in the implementation quality of basic public health services in different regions. It is consistent with the results of RSR ranking. The entropy weight TOPSIS method and RSR fuzzy combination, according to the actual development need to adopt three kinds of proportion analysis, the result found $C_{i}$: $RSR$ = 0.1: 0.9, calculated the top 5 areas are X10, X13, X2, X12, X7 respectively, the bottom 5 areas are X4, X6, X5, X3, X9. (2) $C_{i}$: $RSR$ = 0.5: 0.5, the top 5 areas are calculated as X10, X13, X2, X12 and X1, and the bottom 5 areas are X4, X6, X5, X3 and X9. (3) $C_{i}$: $RSR$ = 0.9: 0.1, and the top 5 areas are calculated as X10, X13, X2, X1 and X12, while the bottom 5 areas are X4, X5, X3, X6 and X9. Although the fuzzy combination of the three proportions is different from the values obtained by the two methods, and the sort is also different, the overall trend is basically the same [28].

However, there are also special phenomena in individual districts. There are differences in the results calculated by the three proportional distributions in X1, X3, X6 and other areas, which indicates that in the above areas, the 23 indicators are unbalanced in management; and when the proportion of the C value is larger, the number of digits higher. This shows that the development of various public health service resources within the region is not balanced, and there are certain extreme values. And due to changes in proportions, weights, etc., the rankings will change. 

## Conclusion

### There are regional differences in the quality of basic public health services, so we should pay attention to the overall improvement and development of services

The results of the study showed that most communities had acceptable quality of basic public health services, with a positive relationship between policy orientation and the degree of economic development and service quality; conversely, areas with the smaller geographical size and population size had poorer quality. This variability is less noticed in previous related studies [29], and the present study adds precisely accordingly. This also indirectly reflects that the basic public health services in China should not only be more solid, but also the content of the work should be adjusted accordingly, adhering to the development policy of "mending the shortcomings and strengthening the weaknesses" of the primary health system. The use of certain community health institutions in this province as the subject of this study is of some relevance, as it reflects the general problem of the quality of public health services in most of central and western China. For the whole central and western regions, we should consolidate the areas with stronger grassroots capacity and support the areas with weaker grassroots capacity with policies and resources, so as to reduce the development gap within and even among provinces, thus promoting the overall improvement of public health service quality and emergency response capacity. Continuously improve infrastructure construction, optimize the allocation of women's and children's health resources, and introduce and train talents in related areas to gradually reduce the differences between regions, improve the quality of women's and children's health care, further clarify service content, processes, requirements, and work indicators, and establish a scientific and standardized process-oriented service system [30].

### There are regional differences in the quality of basic public health services, so we should pay attention to the overall improvement and development of services

In this study, the top 5 weight ratios are the rate of TCM health management for the elderly, the rate of health management for the elderly, the rate of BCG vaccination, the rate of blood sugar control in the managed population, and Vaccination rate of epidemic cerebrospinal meningitis, while the rate of child health management, neonatal Visit rate, regular medication rate for tuberculosis patients, and electronic health record filing rate accounted for relatively low weights. From the results of this study, it can be seen that the health management rate of routine key populations is relatively high, while the health management rate of special key populations such as newborns and pregnant women is low. The low awareness rate of basic public health services has been a long-standing problem since the beginning of public health services. In terms of the management of key populations, regions should pay attention to the health management of the elderly, chronic diseases and children, as well as strengthen the health management of newborns and maternity according to the changes in policies and needs.

Thirdly, in the study, areas such as X10, X13, X2, X1, and X12 have higher grades, and the performance evaluation of basic public health services is higher; while areas such as X9, X6, X5, and X3 are ranked relatively low, and the evaluation of basic public health service performance is lower. Low. In the current study area, the health management of chronic diseases and "family doctor" contracted services for the elderly is not effective, and there is no focus on raising the awareness of their families' supervision. The systematic management of newborns and immunization of children also lacked health promotion and awareness building by professional staff [31]. On the basis of the needs and assessment of the public, an effective dynamic adjustment mechanism is established based on the changes in the health needs of the population. In the future, the organization and implementation of the proposed project and progress scheduling should be developed to continuously improve the regional basic public health services for women's and children's health planning, strengthen the construction of pediatrics in community health centers according to demographic, economic, and spatial elements [32], and improve the timeliness and accessibility of medical services for children.

## Data Availability

The authors confirm that the data supporting the findings of this study are available within the article.

## Footnotes

1 Cilechuan Community Health Service Center, Jinqiao Development Zone Community Health Service Center, Zhaowuda Road Community Health Service Center; Ruyi Community Health Service Center, University West Road Community Health Service Center, (former) University East Road Community Health Service Center, Ulanqab East Road Community Health Service Center, People's Road Community Health Service Center, Zhongzhu Road Community Health Service Center, Yulin Town Health Center, Xihuajie Health Center, Huanghe Shao Health Center, Bayan Health Center, Jinhe Health Center.
